# Charge Redistribution Caused by S,P Synergistically Active Ru Endows an Ultrahigh Hydrogen Evolution Activity of S‐Doped RuP Embedded in N,P,S‐Doped Carbon

**DOI:** 10.1002/advs.202001526

**Published:** 2020-07-20

**Authors:** Xiaoyu Liu, Fan Liu, Jiayuan Yu, Guowei Xiong, Lili Zhao, Yuanhua Sang, Shouwei Zuo, Jing Zhang, Hong Liu, Weijia Zhou

**Affiliations:** ^1^ State Key Laboratory of Crystal Materials Shandong University Jinan 250100 P. R. China; ^2^ Collaborative Innovation Center of Technology and Equipment for Biological Diagnosis and Therapy of Shandong Institute for Advanced Interdisciplinary Research (iAIR) University of Jinan Jinan 250022 P. R. China; ^3^ Guangzhou Key Laboratory for Surface Chemistry of Energy Materials School of Environment and Energy South China University of Technology Guangdong 510006 P. R. China; ^4^ Beijing Synchrotron Radiation Facility Institute of High Energy Physics Chinese Academy of Sciences Beijing 100049 P. R. China; ^5^ University of Chinese Academy of Sciences Beijing 100049 P. R. China

**Keywords:** charge redistribution, hydrogen evolution reaction, bonding regulation, ruthenium phosphide, ternary doping

## Abstract

Water splitting for production of hydrogen as a clean energy alternative to fossil fuel has received much attention, but it is still a tough challenge to synthesize electrocatalysts with controllable bonding and charge distribution. In this work, ultrafine S‐doped RuP nanoparticles homogeneously embedded in a N, P, and S‐codoped carbon sheet (S‐RuP@NPSC) is synthesized by pyrolysis of poly(cyclotriphosphazene‐*co*‐4,4′‐sulfonyldiphenol) (PZS) as the source of C/N/S/P. The bondings between Ru and N, P, S in PZS are regulated to synthesize RuS_2_ (800 °C) and S‐RuP (900 °C) by different calcination temperatures. The S‐RuP@NPSC with low Ru loading of 0.8 wt% with abundant active catalytic sites possesses high utilization of Ru, the mass catalytic activity is 22.88 times than 20 wt% Pt/C with the overpotential of 250 mV. Density functional theory calculation confirms that the surface Ru (−0.18 eV) and P (0.05 eV) are catalytic active sites for the hydrogen evolution reaction (HER), and the according charge redistribution of Ru is regulated by S and P with reverse electronegativity and electron–donor property to induce a synergistically enhanced reactivity toward the HER. This work provides a rational method to regulate the bonding and charge distribution of Ru‐based electrocatalysts by reacting macromolecules with multielement of C/N/S/P with Ru.

## Graphical abstract

Synthesis of electrocatalysts with controllable bonding and charge distribution can optimize charge distribution and phase composition to induce a synergistically enhanced reactivity toward the hydrogen evolution reaction. This work provides a rational method to produce platinum‐like electrocatalysts, and according catalytic active sites are regulated by bonding C/N/S/P in the same molecular structure with Ru.

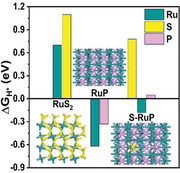

## 1 Introduction

As society come to realize the severity of the energy crisis and environmental pollution, it is urgent to develop effective technologies for clean and sustainable energy form to replace conventional fossil fuels. Electrolytic water has emerged as a promising technology to produce high value‐added and clean hydrogen fuel owing to the merits of strong operability and environmental friendliness. At present, platinum (Pt) noble‐metal‐based^[^ ^1^, ^2^, ^3^ ^]^ catalysts are the state‐of‐the‐art electrocatalysts for hydrogen evolution reaction (HER) owing to the hydrogen absorption Gibbs free energies (Δ*G*_H*_) close to zero, significantly diminish kinetic barrier and enhance rate of electrocatalytic process, but the scarcity and high cost of noble metal severely hinder their large‐scale application. Developing low‐cost and high‐performance electrocatalysts with a low overpotential and practical large current is of great importance in the roadmap of electrolytic water. Currently, the metal‐free^[^ ^4^, ^5^, ^6^, ^7^ ^]^ or nonprecious metal‐based^[^ ^8^, ^9^, ^10^, ^11^ ^]^ electrocatalysts get so much attention. Especially, choosing inexpensive precious metal of Ru^[^ ^12^, ^13^, ^14^ ^]^ and decreasing the loading amount to obtain Pt‐like performance have extraordinary prospect as means of settlement.

Transition metal phosphides, such as Ni_*x*_P,^[^ ^15^, ^16^, ^17^ ^]^ CoP,^[^ ^18^ ^]^ FeP,^[^ ^19^, ^20^ ^]^ and MoP^[^ ^21^, ^22^ ^]^ have presented excellent electrocatalytic properties in HER and OER. Among the less‐expensive precious metals phosphates (e.g., IrP_2_,^[^ ^23^ ^]^ PdP_2_,^[^ ^24^ ^]^ and Rh_2_P^[^ ^25^, ^26^ ^]^), Ru‐based phosphates^[^ ^27^, ^28^ ^]^ as a newly flourished catalysts was the most promising HER catalyst as an alternative to Pt, which has presented excellent catalytic performance in both acid and alkaline electrolytes.^[^ ^29^, ^30^, ^31^ ^]^ It is worth noting that the catalytic performance of electrocatalysts could be enhanced by different anion bonding and heteroatom doping^[^ ^9^, ^32^ ^]^ (e.g., B, N, F, P, or S), which tailored the electronic structure and electrochemical properties of Ru. Qiao selected one couple of the investigated atoms with the most noticeable differences in the charge population (i.e., N and P) as codopants for graphene to maximally activate the adjacent C atom by tailoring its electron donor–acceptor property and consequently enhance its HER activity.^[^ ^33^ ^]^ However, the bonding regulation and charge redistribution of Ru‐based electrocatalysts regulated by different anion bonding and heteroatom doping was rarely reported, which has received much attention.

Herein, we proposed a rational design to synthesize ultrafine S‐doped RuP nanoparticles (<5 nm) embedded in a N,P,S‐codoped carbon sheet matrix (S‐RuP@NPSC) by using the multifunctional highly cross‐linked polymer poly(cyclotriphosphazene‐*co*‐4,4′‐sulfonyldiphenol) (PZS), which is one of the important members of the cyclophosphazene polymers.^[^ ^34^, ^35^, ^36^, ^37^, ^38^ ^]^ The PZS possessed abundant N=P, S=O bonds and functional groups, which have great master in bonding metal ions and limiting the abnormal growth of nanoparticles. More importantly, the PZS with multiple elements of C, N, P, S in the same molecular structure was an ideal model to study the bonding process and reaction mechanism between Ru and C, N, P, S during the pyrolysis process. In addition, PZS used as solid phosphorus source replaced the gas phosphating reaction using the hypophosphites^[^ ^31^ ^]^ and hydrothermal reaction using red phosphorus or trioctylphosphine (TOP)^[^ ^38^, ^39^ ^]^ to avoid producing toxic, flammable polluting tail gas.^[^ ^40^ ^]^ The obtained S‐RuP@NPSC as HER electrocatalyst possessed the high catalytic activity with an overpotential of 92 mV to reach a current density of 10 mA cm^−2^ and rough catalytic stability in alkaline solution. It is worth noting that the S‐RuP@NPSC possessed high utilization of Ru precious metals, the according mass specific activity was 22.88 times than that of 20% Pt/C with the overpotential of 250 mV. This work provides a rational method to regulate the bonding and charge distribution of Ru‐based electrocatalyst by reacting macromolecule with multielement of C/N/S/P with Ru.

## 2 Results and Discussion

**Figure 1:**
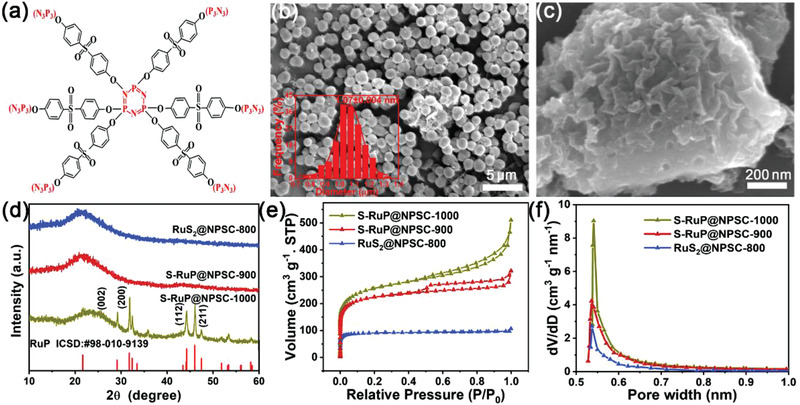
a) The molecular structure of PZS; b,c) SEM images of PZS spheres (b) and samples synthesized at 900 °C (c); the inset of (b) is the particle size distribution, d) XRD pattern, e) nitrogen adsorption–desorption isotherms, and f) pore size distributions of the samples synthesized at different temperatures.

The PZS was macromolecular organic compound, including abundant elements of C, N, P, and S, and the according polymer structure is shown in **Figure** **1**a. The PZS spheres with smooth surface and uniform sizes about 1.07 µm (inset of Figure 1b) were polymerized by hexachlorocyclotriphosphazene (HCCP) and 4,4‐sulfonyldiphenol (BPS), which was observed by scanning electron microscope (SEM) (Figure 1b). Elemental mapping of PZS spheres in Figure S1 (Supporting Information) confirmed the uniformly distributed C, N, P, and S elements in the spheroidal morphology, and the according amounts of C, N, P, and S were 75.49, 3.34, 12.40, and 8.78 at% as shown in Table S1 (Supporting Information), respectively, which were consistent with molecular structure of PZS. However, the obtained PZS spheres were smooth and not conducive to metal ion adsorption. After being pretreated by calcining at 350 °C in the argon atmosphere, the PZS spheres remained spherical morphology (Figure S2, Supporting Information), the hydrophilicity (Figure S3, Supporting Information) was improved which benefited to absorb Ru ions, the according Ru adsorbing capacity of pretreated PZS was about 0.25 wt%. After the calcination at 900 °C under Ar‐H_2_ (10%) mixture gas, the spheres disappeared and nanosheets were observed by SEM in Figure 1c. No obvious particles were observed on the nanosheets, but homogeneous distribution of C, N, P, S, and Ru were detected by elemental mapping in Figure S4 (Supporting Information).

To speculate the formation process of S‐RuP@NPSC, SEM images (Figure 1c and Figure S5, Supporting Information), X‐ray diffractometer (XRD) pattern (Figure 1d), and N_2_ adsorption–desorption isotherms and pore size distribution (Figure 1e,f) of samples synthesized at 800, 900, and 1000 °C were carried out. All samples possessed the random morphologies, implying the collapse of PZS sphere during calcining process. XRD pattern in Figure 1d showed that the carbon bump peaks between 20° and 30° were detected in samples synthesized at 800, 900, and 1000 °C. The XRD pattern of the sample synthesized at 1000 °C showed the characteristic peaks at 29.1°, 32.4°, 44.3°, and 46.0°, which could be well indexed to the (002), (200), (112), and (211) planes of RuP (ICSD: 98‐010‐9139).^[^ ^41^, ^42^ ^]^ However, no diffraction peaks of metal Ru or ruthenium compounds were detected in the samples synthesized at 800 and 900 °C possibly due to the ultrafine nanoparticle. In Figure 1e, there were few mesoporous parts in the samples, but the content of mesoporous relative to microporous and the contribution to the specific surface area were negligible (Figure S6, Supporting Information). As shown in Figure 1e,f, the samples synthesized at 900 and 1000 °C with the abundant microporous structure (average aperture diameter < 2 nm in Figure 1f) possessed the specific surface area of 1147.40 and 1021.13 m^2^ g^−1^, respectively, but the micropores in the sample synthesized at 800 °C was relatively few, as a result, the specific surface area of sample synthesized at 800 °C (357.60 m^2^ g^−1^) was much less than that of 900 and 1000 °C, which implies that the porous structure was mainly formed at above 900 °C, consistent with the N_2_ adsorption–desorption isotherms and pore size distribution of NPSC (Figure S7, Supporting Information) and thermogravimetric analysis–differential scanning calorimetry (TGA‐DSC) results of PZS (Figure S8, Supporting Information).

**Figure 2:**
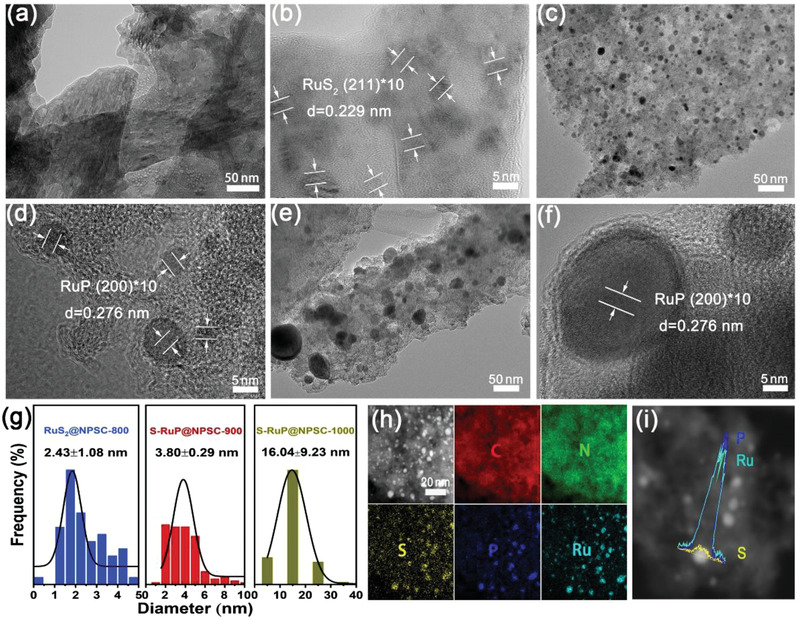
a–f) (HR)TEM images of the samples synthesized at 800 °C (a,b), 900 °C (c,d), 1000 °C (e,f), and g) the respective size distributions. h) Plane and i) linear element distributions of the sample synthesized at 900 °C.

The evolution procedure of S‐RuP@NPSC was further discussed by transmission electron microscopy (TEM) and the according structures are characterized in **Figure** **2**. The nanoparticles on carbon matrix were observed in all samples, however, the according sizes increased with increased calcination temperatures from 800, 900 to 1000 °C, as shown in Figure 2a,c,e. The size distributions are shown in Figure 2g, which were 2.43, 3.80, and 16.04 nm, respectively. As for the samples synthesized at 800 °C, the nanoparticles with crystal lattices of 0.229 nm were detected, corresponding to the (211) crystal plane of RuS_2_ (Figure 2b), implying the Ru species was vulcanized by PZS at 800 °C. When the temperature increased to 900 and 1000 °C, the clear crystal lattices with interplanar distance of 0.276 nm corresponding to the (200) crystal plane of RuP were detected. However, the RuP nanoparticles synthesized at 1000 °C possessed the higher crystallinity and larger particle size, which was consistent with XRD result in Figure 1d.

The energy‐dispersive spectroscopy (EDS) element mapping showed that C, N, P, S elements were homogeneously distributed on the whole matrix in all samples, as shown in Figure S9 (Supporting Information), Figure 2h, and Figure S10 (Supporting Information). The element mapping of C, N, P, S, and Ru in the sample synthesized at 800 °C were indistinguishable due to the too small size of RuS_2_ nanoparticles. In addition, P, S, and Ru elements presented concentrated distribution at tiny nanoparticles in the samples synthesized at 900 and 1000 °C, implying that P and S elements were easier to bond to Ru, but N element was more prone to doping into carbon. It was important that the line scanning of element mapping in Figure 2i detected that the molar ratio between Ru and P was 1:1 and the S content was slight. Hence, the nanoparticles on NPSC were speculated to be S‐doped RuP. It was not difficult to see that there was a stronger interaction involving Ru and P, S, and no bonding between Ru and N. Therefore, we could draw the preliminary speculation that the RuS_2_ on NPSC was synthesized at 800 °C (denoted as RuS_2_@NPSC‐800) and S‐RuP on NPSC was synthesized at 900 and 1000 °C (denoted as S‐RuP@NPSC‐900 and S‐RuP@NPSC‐1000, respectively).

**Figure 3:**
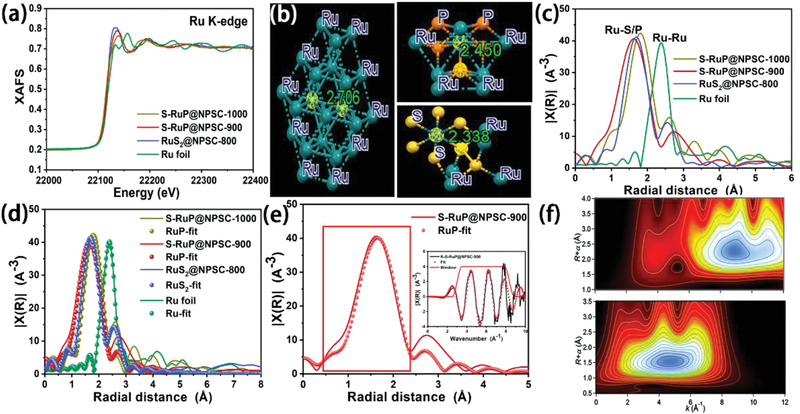
a) Normalized XANES spectra at Ru K‐edge, b) the crystallographic data of Ru, RuP, and RuS_2_, c) FT‐EXAFS, d,e) FT‐EXAFS fitting spectra at Ru K‐edge of Ru foil and RuS_2_@NPSC‐800, S‐RuP@NPSC‐900, S‐RuP@NPSC‐1000; the inset of (e) shows the k space fitting curve; and f) WT‐EXAFS of the Ru foil and S‐RuP@NPSC‐900.

To further determine the structure and composition of the bonding in the samples. The surface chemical states of RuS_2_@NPSC‐800, S‐RuP@NPSC‐900, and S‐RuP@NPSC‐1000 were investigated by X‐ray photoelectron spectroscopy (XPS) in Figure S11 (Supporting Information). The elements of C, N, S, and P were detected in XPS survey spectra, but no obvious peaks of Ru due to the low loading, which were 0.73, 0.80, and 0.85 wt% measured by inductively coupled plasma atom emission spectrometry (ICP) (Table S2, Supporting Information) for RuS_2_@NPSC‐800, S‐RuP@NPSC‐900, and S‐RuP@NPSC‐1000, respectively. The average ruthenium valence state and the electronic structure of Ru foil, RuS_2_@NPSC‐800, S‐RuP@NPSC‐900, and S‐RuP@NPSC‐1000 were further characterized by synchrotron‐radiation‐based hard X‐ray absorption fine structure (XAFS) as shown in **Figure** **3**. In a nutshell, the oxidation state of Ru can be represented visually through the absorption threshold position of Ru K‐edge in Figure 3a. The energy of pre‐edges of RuS_2_@NPSC‐800, S‐RuP@NPSC‐900, and S‐RuP@NPSC‐1000 were lower than that of metallic Ru foil, indicating the oxidation state of the Ru synthetic samples decreased.

The crystallographic data of Ru, RuP, and RuS_2_ in Figure 3b showed that the distances of Ru—Ru, Ru—S, and Ru—P were 2.71, 2.34, and 2.45 Å, respectively. It is worth noting that the position of the peak obtained by the Fourier transform was not the actual bond length, which was generally shorter than real bond length. Figure 3c–e shows a comparison of the Fourier transforms of the extended X‐ray absorption fine structure (FT‐EXAFS) at the Ru K‐edge of the different samples to extract the structural parameters. As shown in Figure 3c, the prominent peak for Ru foil at around 2.50 Å corresponded to the Ru—Ru bond and the prominent peak for RuS_2_@NPSC‐800, S‐RuP@NPSC‐900, and S‐RuP@NPSC‐1000 at around 1.81 Å corresponded to the Ru—S/P bond.^[^ ^16^, ^43^, ^44^ ^]^ The EXAFS fitting in Figure 3d was performed to extract the structural parameters, it was clearly seen that RuS_2_@NPSC‐800 was well matched with the RuS_2_, the S‐RuP@NPSC‐900, and S‐RuP@NPSC‐1000 were quite well fit with RuP. The EXAFS fitting and according k space fitting curve of S‐RuP@NPSC‐900 are shown in Figure 3e, and these excellent fit results revealed the according phase of S‐RuP@NPSC‐900 was RuP, which was well consistent with the XRD and (HR)TEM. In addition, the Ru—P distance in S‐RuP@NPSC‐900 was slightly smaller than that in S‐RuP@NPSC‐1000. Considering the crystallographic data of RuP and RuS_2_, the increase of Ru—P distance in S‐RuP@NPSC‐1000 confirmed that the S doping amount in S‐RuP decreased at higher calcination temperature.

Wavelet transform (WT) was also thought to be a strong support for FT. Owing to the powerful resolution in both k and R spaces, WT‐EXAFS was generally employed to probe atomic coordination. The Ru K‐edge WT‐EXAFS of the Ru foil and S‐RuP@NPSC‐900 are illustrated in Figure 3f. The WT signal related to Ru—Ru contribution was detected in Ru foil, intensity maximum at 8.80 Å^−1^. The WT contour plot of the S‐RuP@NPSC‐900 displayed only one intensity maximum at 4.80 Å^−1^, which could be assigned to the Ru—P coordination. Based on the EXAFS fitting and WT‐EXAFS, it was thought that the Ru ions were firstly vulcanized at about 800 °C, followed by further phosphating and S existed in a doping form to transform to S‐RuP at 900 and 1000 °C. Therefore, the bonding between Ru and N, P, S elements in PZS was regulated by calcination temperature to control the phase composition of S‐doped RuP on N,P,S‐doped carbon.

**Figure 4:**
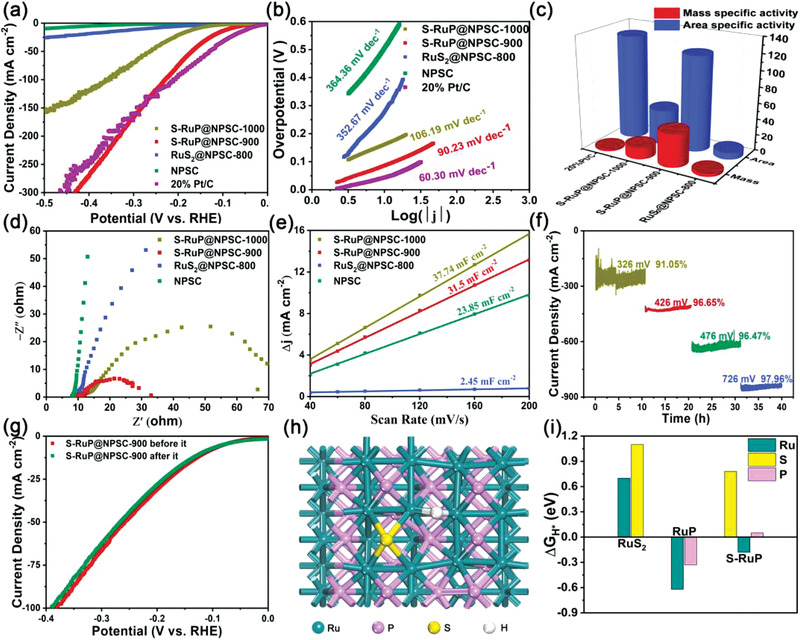
a) LSV curves recorded at 5 mV s^−1^, b) corresponding Tafel plots of RuS_2_@NPSC‐800, S‐RuP@NPSC‐900, S‐RuP@NPSC‐1000, NPSC, and 20% Pt/C, c) comparison with respect to the area and mass specific activity with the overpotential of 250 mV (area specific activity: mA cm^−2^ and mass specific activity: A mg^−1^), d) Nyquist plots, e) electrochemical surface area of Ru‐RuS_2_@NPSC‐800, S‐RuP@NPSC‐900, S‐RuP@NPSC‐1000, and NPSC, f) time‐dependent current density curve of S‐RuP@NPSC‐900 under static overpotentials in 1 m KOH, g) LSV curves before and after *i*–*t* testing of S‐RuP@NPSC‐900, h) optimized structure of S‐doped RuP and H* adsorption on Ru, and i) the Gibbs free energy of H* adsorption (Δ*G*_H*_) on various sites.

The HER activities of RuS_2_@NPSC‐800, S‐RuP@NPSC‐900, S‐RuP@NPSC‐1000, NPSC, and 20% Pt/C were tested by a typical three‐electrode system in alkaline solution (1 m KOH). The HER polarization curve in **Figure** **4**a showed that the S‐RuP@NPSC‐900 possessed a much lower overpotential of 92 mV to achieve the current density of 10 mA cm^−2^ than those of RuS_2_@NPSC‐800 (300 mV) and S‐RuP@NPSC‐1000 (170 mV) and NPSC (506 mV). In addition, the S‐RuP@NPSC‐900 could achieve a higher current density of 320.09 mA cm^−2^ than that of 20% Pt/C (310.34 mA cm^−2^) with the overpotential of 450 mV. The above results confirmed that the formation of the ultrafine S‐doped RuP nanoparticles into N/P/S‐doped carbon was fatal to improve the HER activity of S‐RuP@NPSC‐900. The HER performances of samples were also tested in 1 m PBS and 0.5 m H_2_SO_4_, the S‐RuP@NPSC‐900 also exhibited the efficient HER performance in neutral and acidic environment, as shown in Figure S12 (Supporting Information). To further explore the HER mechanism, the according Tafel plots and slopes are shown in Figure 4b. The Tafel slope value of S‐RuP@NPSC‐900 (90.23 mV dec^−1^) was smaller than those of NPSC (364.36 mV dec^−1^), RuS_2_@NPSC‐800 (352.67 mV dec^−1^), and S‐RuP@NPSC‐1000 (106.19 mV dec^−1^), and was larger than that of 20 wt% Pt/C (60.30 mV dec^−1^), confirming the desorption of H* (either the ion and atom reaction) was the rate‐determining step in HER reaction process (Heyrovsky–Volmer reaction).^[^ ^9^, ^32^, ^45^, ^46^ ^]^

It was worth emphasizing that RuS_2_@NPSC‐800, S‐RuP@NPSC‐900, and S‐RuP@NPSC‐1000 possessed the ultralow loading amounts of Ru measured by ICP, which were 0.73, 0.8, and 0.85 wt%, respectively. Area and mass specific activity of RuS_2_@NPSC‐800, S‐RuP@NPSC‐900, S‐RuP@NPSC‐1000, and 20 wt% Pt/C with the overpotential of 250 mV were calculated by the content of precious metals of Ru and Pt. As shown in Figure 4c, the area specific activity of S‐RuP@NPSC‐900 (118.36 mA cm^−2^) was similar to that of 20% Pt/C (129.34 mA cm^−2^), but the according mass specific activity of S‐RuP@NPSC‐900 (41.43 A mg^−1^) was 22.88 times than that of 20% Pt/C (1.81 A mg^−1^). Undoubtedly, the S‐RuP@NPSC‐900 possessed overwhelming advantage in current density after normalized by Ru loading, which revealed the high utilization of Ru. Comparing with current relevant literature with the overpotential of 50 mV, the HER mass specific activity of S‐RuP@NPSC‐900 (871.50 A g^−1^) with an ultralow loading of Ru (0.8 wt%) was larger than those of the recently reported Ru‐based HER electrocatalysts, such as RuP_2_@NPC (64.38 A g^−1^),^[^ ^47^ ^]^ Ru‐Ru_2_PΦNPC (2.96 A g^−1^),^[^ ^40^ ^]^ RuP/CC (38.40 A g^−1^), RuP_2_/CC(7.69 A g^−1^),^[^ ^41^ ^]^ and the ultrasmall Ru_2_P NPs@GO (765.63 A g^−1^),^[^ ^48^ ^]^ which showed that S‐RuP@NPSC‐900 was a promising and competitive HER catalyst.

The Nyquist plots of different samples are shown in Figure 4d. The electron transfer resistance (*R*_ct_) value (20.86 Ω) of S‐RuP@NPSC‐900 was lower than those of NPSC (over 1000 Ω), RuS_2_@NPSC‐800 (459 Ω), and S‐RuP@NPSC‐1000 (60.01 Ω), indicating that it had the fastest electrochemical reaction rate for HER. The Nyquist plots of S‐RuP@NPSC‐900 and the fitting of equivalent circuit diagram are shown in Figure S13 (Supporting Information), the *R*_ct_ value of S‐RuP@NPSC‐900 decreased from 506.80 to 20.86 Ω with the increased overpotentials from 100 to 250 mV, implying that there was a quick HER kinetics toward the interface between electrolyte and electrode.

The double‐layer capacitances (*C*_dl_) were measured by cyclic voltammograms (CVs) to evaluate the electrochemical active surface area (ECSA) of all the samples, as shown in Figure 4e and Figure S14 (Supporting Information). The S‐RuP@NPSC‐900 possessed a large electrochemical area of 31.5 mF cm^−2^, close to the S‐RuP@NPSC‐1000 (37.73 mF cm^−2^), which was larger than NPSC (23.85 mF cm^−2^) much larger than that of RuS_2_@NPSC‐800 (2.45 mF cm^−2^), which was consistent with nitrogen adsorption–desorption isotherms result (Figure 1e), implying that the large electrochemical area was mainly attributed to the high specific surface area of NPSC. However, the S‐RuP@NPSC‐1000 possessed the largest electrochemical area but it did not have the best catalytic activity for HER, implying that both the high utilization of precious Ru catalytic sites and large electrochemical area determined the high HER performance of S‐RuP@NPSC‐900. In addition, it was clearly observed that after being calibrated by ECSA, S‐RuP@NPSC‐900 still exhibited the optimal HER activity among all the samples, indicating the highest intrinsic catalytic activity of S‐RuP@NPSC‐900 (Figure S15, Supporting Information).

The large current density and long‐term stability were important parameters of HER for industrial application. As shown in Figure 4f, the S‐RuP@NPSC‐900 electrode had undergone a HER stability test for every 10 h with current densities of 200, 400, 600, and 800 mA cm^−2^, respectively, and the according attenuations at the static overpotentials were 9% at 326 mV, 4% at 426 mV, 3% at 476 mV, and 2% at 726 mV. The negligible attenuation in polarization curves of S‐RuP@NPSC‐900 before and after *i*–*t* testing further confirmed the robust HER stability in 1 m KOH, as shown in Figure 4g. The above results unambiguously demonstrated that the S‐RuP@NPSC‐900 possessed long‐term catalytic stability and resistance in strong corrosive environment, making it a promising catalyst for potential industrial applications.

The above results indicated that the S‐RuP was the main catalytic active component for HER and the NPSC was functioned for the electron transfer and electrolyte ion diffusion. In order to further understand the intrinsic HER catalytic activity and charge distribution of RuS_2_ and S‐RuP, density functional theory (DFT) calculations utilizing Vienna Ab initio Simulation Package (VASP) were performed to explore the geometric properties and original active sites, in which the projected augmented wave method and Perdew–Burke–Ernzerhof (PBE) exchange correlation were used. The schematic structure and according Gibbs free energy of H* adsorption (|Δ*G*_H*_|) of various sites, including Ru, S sites in RuS_2_, Ru, P sites in RuP, and Ru, P, S sites in S‐RuP, are shown in Figure 4h,i and Figures S16–S18 (Supporting Information). The optimal value of |Δ*G*_H*_| should be close to zero. The positive Δ*G*_H*_ implied that the formation of intermediate H* was the rate‐determining step for HER. While, the negative Δ*G*_H*_ implied the desorption of H* to form H_2_ was the rate‐determining step for HER.^[^ ^49^ ^]^ We could figure out that the Δ*G*_H*_ value of Ru, S sites in RuS_2_ were 0.7 and 1.1 eV, implying too weak H* adsorption and the poor intrinsic HER activity, consistent with electrochemical result in Figure 4a and previous reports.^[^ ^50^, ^51^, ^52^ ^]^ It is worth noting that the Δ*G*_H*_ of RuP was opposite to that of RuS_2_, and the calculated Δ*G*_H*_ values of Ru and P sites were −0.62 and −0.33 eV, respectively. After the S doping into RuP, the calculated Δ*G*_H*_ values of Ru and P sites in S‐RuP were −0.18 and 0.05 eV, which were significantly reduced, and close to zero. However, the Δ*G*_H*_ value of 0.78 eV for S site in S‐RuP was still high, implying the Ru and P sites were the HER catalytic active sites in S‐RuP, which were regulated by S doping. The DFT calculation confirmed that the S and P with reverse electronegativity could lead to a unique electron‐donor property in S‐RuP, which has been reported as synergistic coupling effect between two heteroatoms and carbon, consequently, largely boost its oxygen reduction reaction (ORR)^[^ ^53^, ^54^ ^]^ and HER activity.^[^ ^33^, ^55^ ^]^

## 3 Conclusion

In summary, ultrafine S‐doped RuP nanoparticles with size less than 5 nm and low Ru loading (0.80 wt%) homogeneously embedded in the N, P and S‐codoped carbon sheet matrix (S‐RuP@NPSC) with large specific surface area of 1147.40 m^2^ g^−1^ was successfully synthesized by pyrolysis of PZS as C, N, P, and S sources. There was a stronger interaction between Ru and P, S and no bonding between Ru and N. The bonding between Ru and P, S elements in PZS was regulated by controlling calcination temperature to obtain the controllable phase composition of RuS_2_@NPSC (800 °C) and S‐RuP@NPSC (900 and 1000 °C). The obtained S‐RuP@NPSC‐900 as electrocatalyst possessed the platinum‐like activity for HER and excellent long‐term stability in alkaline solution (1 m KOH), and could achieve a current density of 10 mA cm^−2^ with an overpotential of 92 mV. The S‐RuP@NPSC‐900 possessed high utilization of Ru metal, and the according mass catalytic activity of S‐RuP@NPSC‐900 was 22.88 times than that of 20 wt% Pt/C with the overpotential of 250 mV. Theoretical calculation confirmed that the surface Ru atom (−0.18 eV) and P atom (0.05 eV) were HER catalytic active sites in S‐RuP. The S and P heteroatoms with reverse electronegativity and electron‐donor property could coactivate the Ru atom by affecting its charge distribution to induce a synergistically enhanced reactivity toward HER. This work provided a rational and controllable method to produce Ru‐based electrocatalyst with regulated catalytic active sites by bonding regulation and charge redistribution.

## Conflict of Interest

The authors declare no conflict of interest.

## Supporting information

Supporting Information
