# Supplementary material for: Charge Redistribution Caused by S,P Synergistically Active Ru Endows an Ultrahigh Hydrogen Evolution Activity of S‐Doped RuP Embedded in N,P,S‐Doped Carbon
Source: Adv Sci (Weinh). 2020 Jul 20;7(17):2001526. doi: 10.1002/advs.202001526 (PMC7507474; doi:10.1002/advs.202001526)
Supplement: Supplementary file 1 — Supporting Information [file ADVS-7-2001526-s001.pdf]

Copyright WILEY-VCH Verlag GmbH & Co. KGaA, 69469 Weinheim, Germany, 2020.

Supporting Information

S, P Synergistically Active Ru Caused Charge Redistribution Endows a Ultrahigh Hydrogen Evolution Activity of S-doped RuP Embedded in N, P, S-doped Carbon

Xiaoyu Liu, Fan Liu, Jiayuan Yu, Guowei Xiong, Lili Zhao, Yuanhua Sang, Shouwei Zuo, Jing Zhang, Hong Liu, Weijia Zhou**

Experimental Section

Chemicals: Hexachlorocyclotriphosphazene (HCCP), 4,4'-sul-fonyldiphenol (BPS), ruthenium(III) chloride hydrate ($RuCl_3{\cdot}3H_2O$), sulfuric acid (H_2SO_4), potassium hydroxide (KOH), sodium dihydrogen phosphate (NaH_2PO_4), disodium hydrogen phosphate (Na_2HPO_4) conductive carbon black (XC-72) were of analytical grade and used without further purification. Deionized water was supplied with a Barnstead Nanopure Water System. Mixed gases of argon and hydrogen (Ar-H_2, 10 vol% H_2) and argon were obtained from Jinan DeYuan Gases Co. Ltd.

Synthesis of the poly (cyclotriphosphazene-co-4, 4'-sulfonyldiphenol) (PZS) spheres

Synthesis of the poly (cyclotriphosphazene-co-4, 4'-sulfonyldiphenol) (PZS) spheres were mainly by one-step precipitation polymerization, in a typical polymerization system, 2 mL triethylamine (TEA) was added to a solution of hexachlorocyclotriphosphazene (HCCP) (0.1 g) and 4,4'-sul-fonyldiphenol (BPS) (0.225 g) in acetone (50 mL). The reaction mixture was stirred in an ultrasonic bath (100 W, 40 kHz) at 30-40 ℃ for 4 hours. After ultrasonic bath, milky white solution was obtained, filtered and then the precipitates were washed several times with ethanol and deionized water, respectively. Finally, the filtered solid was dried under vacuum to yield PZS spheres.

Synthesis of the S doped RuP nanoparticles@N, P, S doped carbon materials (S-RuP@NPSC)

Firstly, PZS spheres were pre-calcined at 350 ℃ in an argon atmosphere to increase the hydrophilicity for the next step of adsorption of ruthenium (Ru), the white powder turned to yellow after the pretreatment. 0.1 g of the pre-pretreated precursor was dispersed into 20 mL deionized water, then 0.001 g of ruthenium (III) chloride was added in the PZS solution under magnetic stirring for 4 h at room temperature, which aimed ruthenium ions to adsorb on the surface of PZS sphere. Finally, it was centrifuged at 7000 rpm for 5 min to remove excess ruthenium ions, Subsequently the centrifugal mixture was desiccated by the vacuum freeze-drying to obtain the Ru adsorbed PZS (Ru@PZS). After drying, the obtained Ru@PZS was placed at the center of the quartz tube inside a tubular furnace and thermally treated at 900 ^{o}C for 2 h under Ar-H_2 (10%) mixture gas flow of 50 sccm, and then cooled to ambient temperature under Ar-H_2 (10%). After the calcination, the S doped RuP nanoparticles embedded into N, P, S co-doped carbon was obtained, which was denoted as S-RuP@NPSC-900. Considering the different phases were synthesized, the samples with different calcination temperatures were denoted as RuS_2@NPSC-800, S-RuP@NPSC-900 and S-RuP@NPSC-1000.

Characterization

Phase compositions of the as-made materials were performed on a powder X-ray diffractometer (Cu Kα, λ = 0.15406 nm, Bruker D8 Advance, Germany). Morphologies of the as-made materials were identified by a field emission scanning electron microscope (FESEM, MERLIN Compact, Carl Zeiss) and a transmission electron microscope (TEM, a JEM-2100F Field Emission Electron Microscope, JPN) at an acceleration voltage of 200 kV. X-ray photoelectron spectroscopic (XPS) measurement was performed using a PHI X-tool instrument (Ulvac-Phi). Brunauer-Emmet-Teller (BET) specific surface area (SSA) and pore size distribution (PSD) were obtained by using Pierre Kubox 2000 specific surface aperture analyser (kubo-x2000) with nitrogen adsorption at 77 K using the Barrett-Joyner-Halenda (BJH) and Dubinin-Radushkevich (DR) method. The differential thermal analyzer and

thermal gravimetric (TGA-DSC) was performed with a TGA/DSC Mettler-Toledo thermal analyzer from 30 to 1000 ℃ at a heating rate of 5 ℃/min in a stream of Nitrogen (50 mL/min). XAFS measurements at the Ru K-edge in both transmission (for Ru foil) and fluorescence (for samples) mode were performed at Beijing Synchrotron Radiation Facility (beamline 1W1B station), China. The content of Ru element was determined by inductively coupled plasma optical emission spectrometer (Agilent ICP-OES 730).

Electrochemistry

Electrochemical measurements were performed with an electrochemical workstation (CHI 760E, Chenhua Instruments Inc.) in 0.5 M H_2SO_4, 1 M phosphate buffer (BPS) and 1 M KOH, Hg/Hg_2Cl_2 electrode (SCE, saturated KCl), Hg/HgO electrode (Hg/HgO, 1 M KOH) and Ag/AgCl electrode and carbon rod were used as the reference and counter electrode, separately. 5 mg sample was dispersed in 1 mL of 4:1 (v: v) water/ethanol mixed solvents along with 50 μL Nafion solution, and the mixture was sonicated enough. Then, 5 μL mixture was dripped over the glassy-carbon electrode of 0.07 cm^2 at a catalyst loading of 0.357 mg cm^{-2}. Polarization curves were achieved by sweeping the potential from 0 to -0.5 V vs. RHE at a sweep rate of 5 mV/s. Unless specifically mentioned, all the electrochemical measurements were iR-corrected. AC impedance was detected with a frequency range and an amplitude of 5 mV from 0.01 Hz to 100 kHz. Electrochemical impedance spectroscopy (EIS) measurements were detected with a frequency range and amplitude of 200 mV from 0.01 Hz to 100 kHz. The main arc in electrochemical impedance spectroscopy (EIS) spectra was matched utilizing a simplified Randles equivalent circuit, which was composed of a resistance (Rs) in series with a parallel arrangement of a charge-transfer resistance (Rct) and a constant phase element (CPE), and the fitting parameters were appraised through the Levenberg-Marquardt minimization procedure. Cyclic voltammetry (CV) was applied to measure the electrochemical double layer capacitance at non-faradaic potentials as another way to reckon the efficient electrochemical active area of HER.

DFT Theoretical calculations

All the spin-polarized computations were performed by using Vienna *ab-initio* simulation package (VASP). The ion-electron interactions were described by the projector augmented wave method and the general gradient approximation in the Perdew-Burke-Ernzerhof (PBE) form was used. During the structure relaxation, the convergence criterion was set to 0.03 eV/Å and 10^{-5} eV for the residual force and energy, respectively. Brillouin zones were sampled by a Monkhorst-Pack k-point mesh with a 3 × 3 × 1 k-point grid. To avoid the interaction between two periodic units, a vacuum space of 20 Å was employed.

The free energy change (ΔG) of each elementary reaction was calculated as

$$\Delta G = \Delta E + \Delta E_{\mathrm{ZPE}} - T\Delta S$$

where ΔE, E_{ZPE}, T and S is the reaction energy difference, zero-point energies, temperature and entropy, respectively.

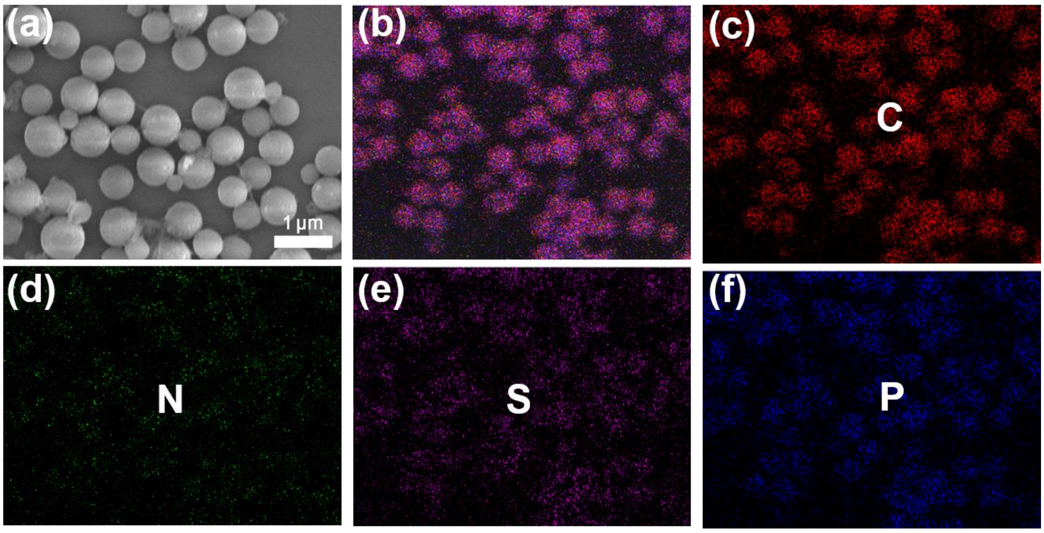

Figure S1. (a) SEM image and EDS Elemental mapping (b) overlap, (c) C, (d) N, (e) S, (f) P of PZS spheres.

Table S1. The element content analysis spectrum of PZS spheres.

Elt.	Line	Intensity (c/s)	Conc.	Units	Error 2-sig	MDL 3-sig	
**C**	Ka	52.31	75.492	wt.%	6.091	7.058	
**N**	Ka	9.987	3.337	wt.%	0.678	1.012	
**P**	Ka	42.14	12.390	wt.%	1.098	1.299	

**S**	Ka	26.28	8.781	wt.%	0.988	1.172	
			100.000	Wt.%			Total

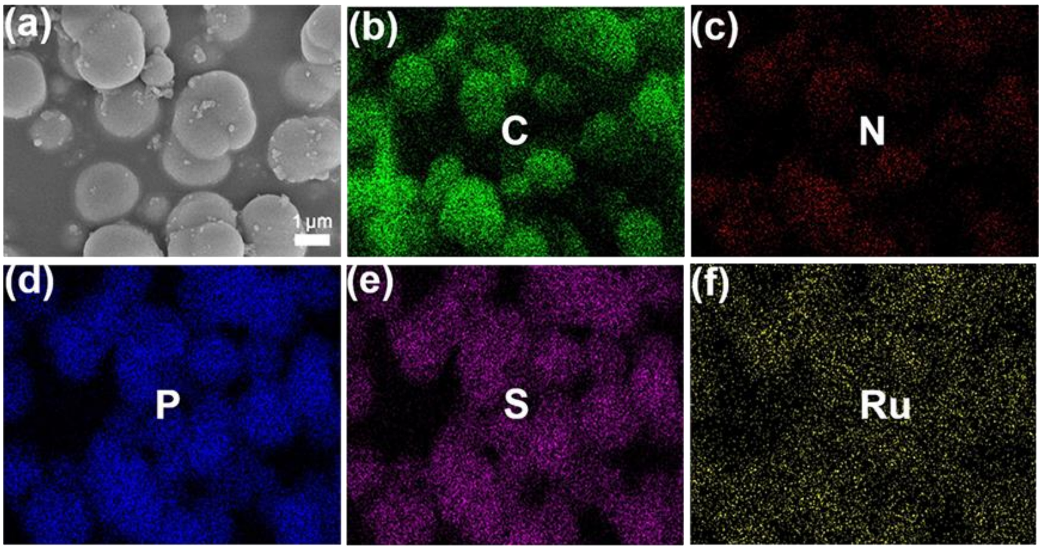

Figure S2. (a) SEM image and EDS Elemental mapping (b) C, (c) N, (d) P, (e) S , (f) Ru of Ru^{3+} adsorbed on the surface of 350 °C pretreated PZS spheres.

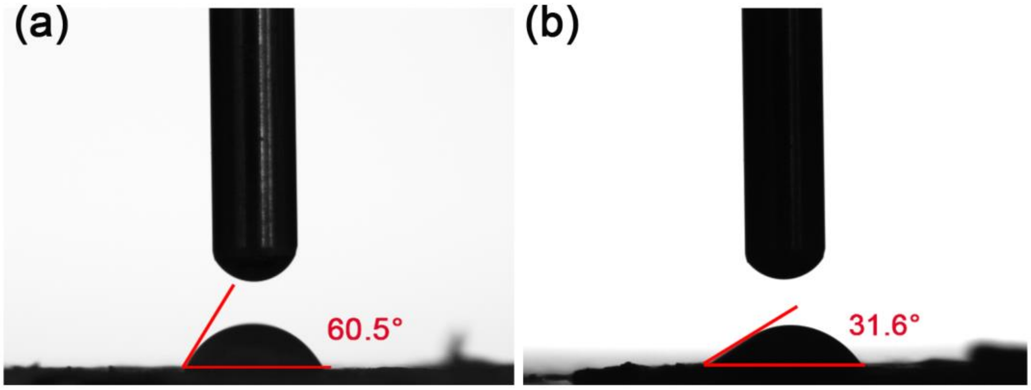

Figure S3. Contact angle of (a) PZS spheres, (b) PZS spheres pretreated by calcining at 350 °C.

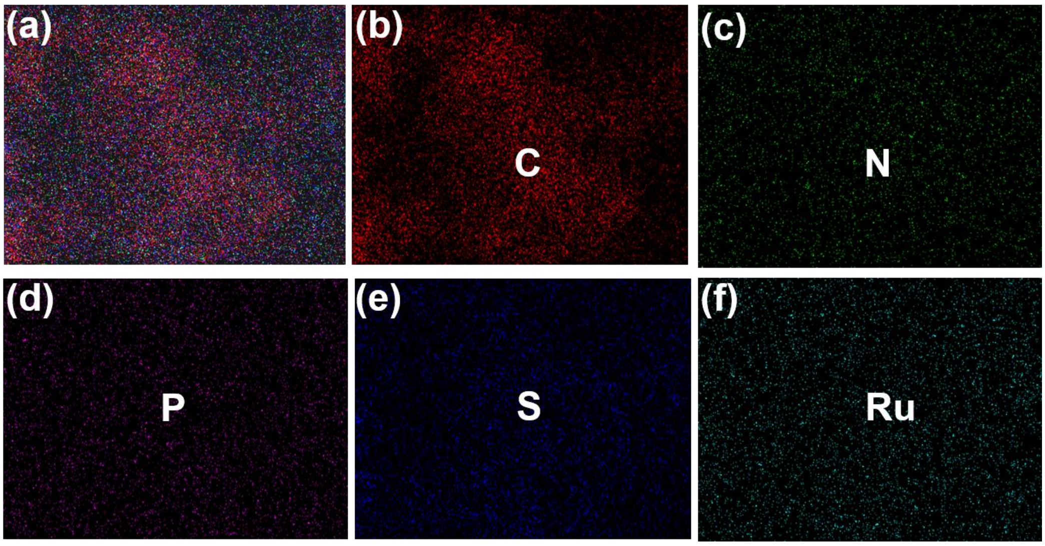

Figure S4. EDS Elemental mapping (a) overlap, (b) C, (c) N, (d) P, (e) S , (f) Ru of S-RuP@NPSC-900.

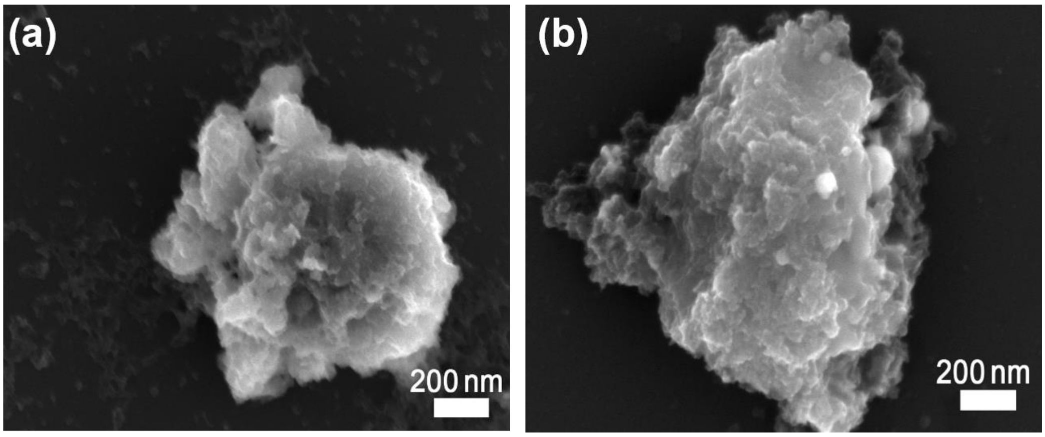

Figure S5. SEM images of (a) RuS_2@NPSC-800 and (b) S-RuP@NPSC-1000.

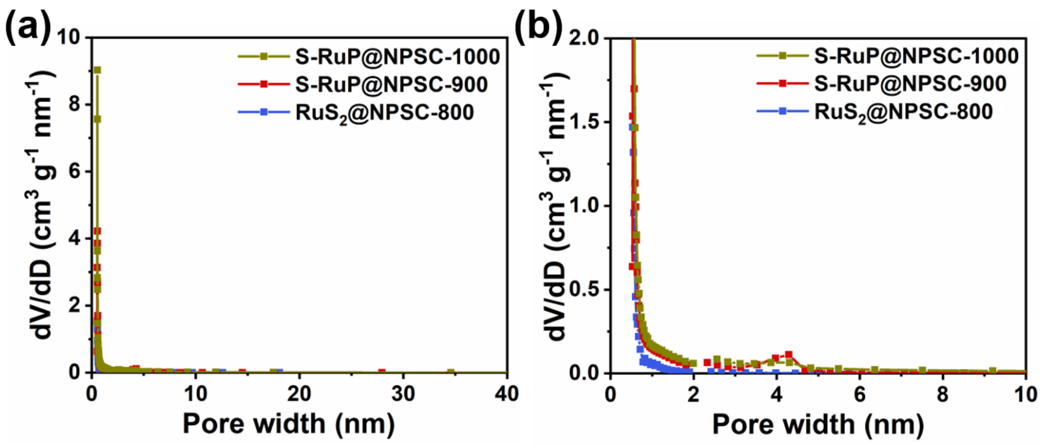

Figure S6. Pore size distributions of the samples synthesized at different temperatures.

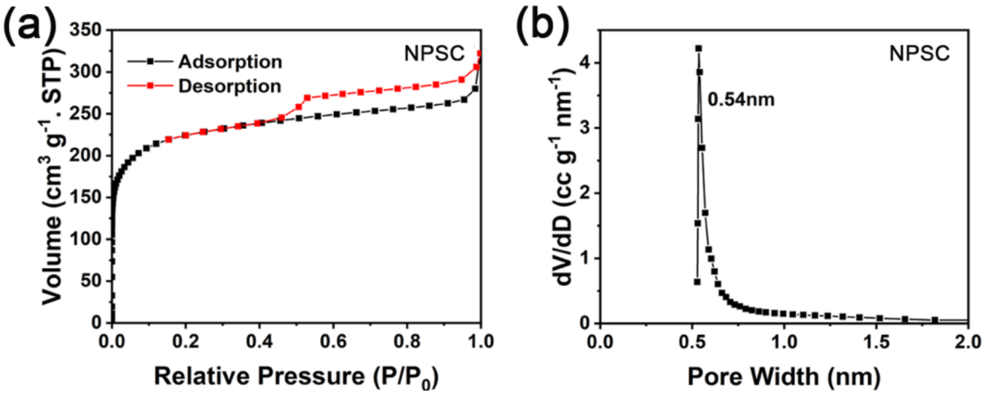

Figure S7. N_2 adsorption-desorption isotherms and pore size distribution of NPSC

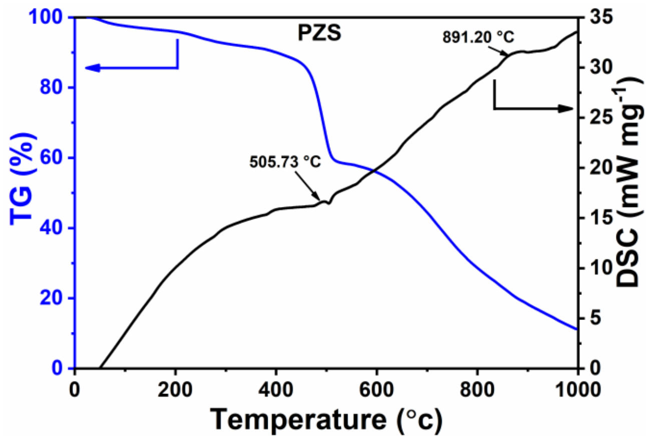

Figure S8. TG-DSC curve of PZS.

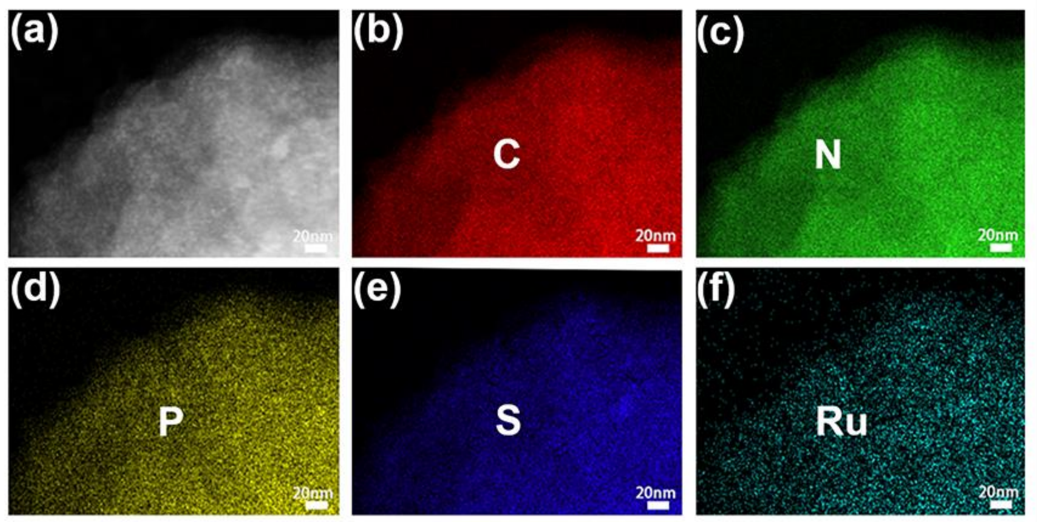

Figure S9. (a) HRTEM imagine and EDS element mapping (b) C, (c) N, (d) P, (e) S , (f) Ru of RuS_2@NPSC-800.

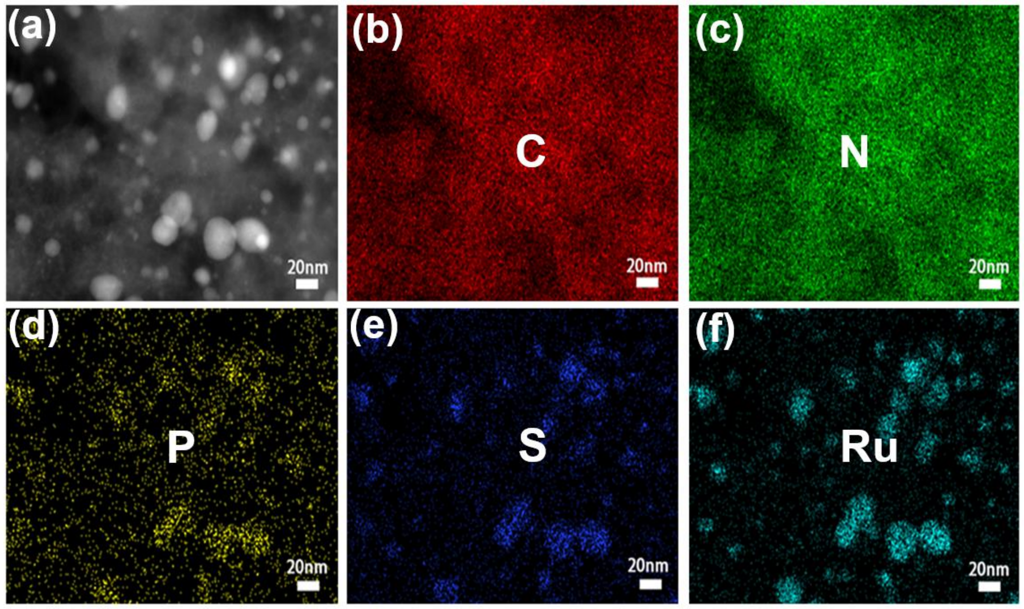

Figure S10. (a) HRTEM imagine and EDS element mapping (b) C, (c) N, (d) P, (e)S , (f) Ru of S-RuP@NPSC-1000.

Table S2. The Ru contents in different electrocatalysts measured by ICP-OES.

Electrocatalyst	Ru Content (wt%)
RuS_2@NPSC-800	0.73
S-RuP@NPSC-900	0.80
S-RuP@NPSC-1000	0.85

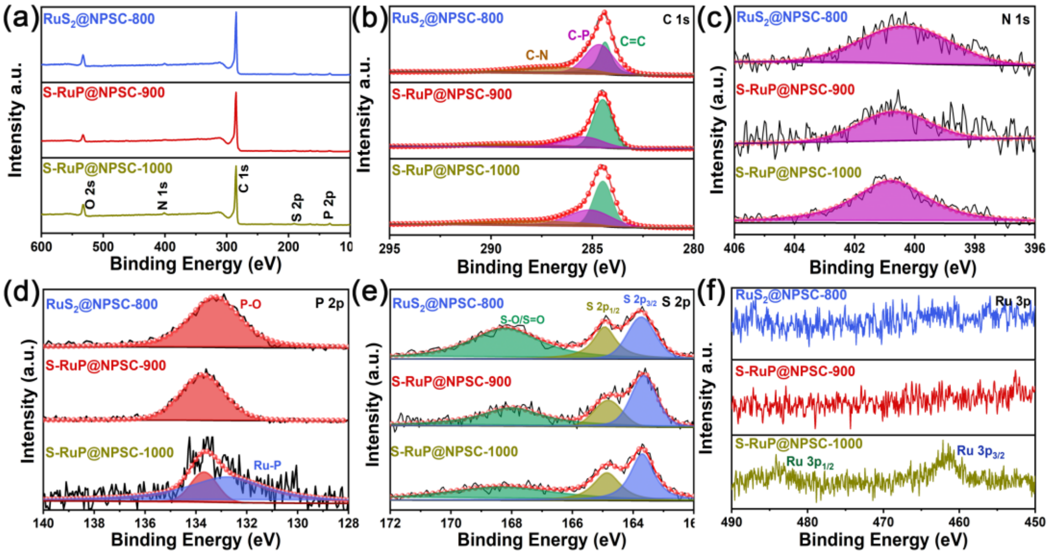

Figure S11. XPS full spectra (a) and High-resolution spectra of (b) C 1s (c) N 1s, (d) P 2p, (e) S 2p and (f) Ru 3p of RuS_2@NPSC-800, S-RuP@NPSC-900 and S-RuP@NPSC-1000.

The surface chemical states of RuS_2@NPSC-800, S-RuP@NPSC-900, S-RuP@NPSC-1000 were further investigated by X-ray photoelectron spectroscopy (XPS). All of completed spectrums were shown in **Figure S11a**, in which five peaks are observed corresponding to C,

N, O, S and P, but no obvious peaks of Ru due to the low loading. The ICP were used to confirm the Ru content of RuS_2@NPSC-800, S-RuP@NPSC-900, S-RuP@NPSC-1000, the Ru content were 0.73 wt%, 0.8 wt% and 0.85 wt%. High-resolution XPS spectra of C1s **(Figure S11b)** showed three separated peaks at 284.37 eV for C=C, 284.65 eV for C-P, 286.92 eV for C-N, and it could see that the intensity of C-P gradually decreased as the calcination temperature increasing. High-resolution The peak corresponding to P-O at 133.68 eV was detected in RuS_2@NPSC-800, S-RuP@NPSC-900 and S-RuP@NPSC-1000 in **Figure S11d**, the peak located at 132.73 eV corresponding to Ru-P gradually formed, when the calcination temperature reached 1000 ℃, strong electron interactions involving Ru and P are revealed in S-RuP@NPSC-1000, only P-O bonds at 133.68 eV and Ru-P bond at 132.73 eV were detected. The C-P decreasing and Ru-P gradually formed, which was consistent with the formation of new RuP phase in XRD and (HR)TEM. XPS spectra of N1s **(Figure S11c)** and S2p **(Figure S11e)** have little change among RuS_2@NPSC-800, S-RuP@NPSC-900, S-RuP@NPSC-1000, which implying most of N and S were doped into carbon. As illustrated in **Figure S11e**, the high-resolution S 2p spectrum had three separated peaks at 163.65 eV for S $2p_{3/2}$, 164.85 eV for S $2p_{1/2}$, 168 eV for S-O/S=O which were derived from the valency bond state of S in the polymerized monomer (HCCP). The high-resolution Ru 3p spectrum was shown in **Figure S11f**, it could see that there were no obvious peaks of Ru in RuS_2@NPSC-800, S-RuP@NPSC-900 due to the low loading of Ru. When the calcination temperature reached 1000 ℃, little peak corresponding to Ru $3p_{1/2}$ at 484.60 eV and Ru $3p_{3/2}$ at 462.00 eV was detected.

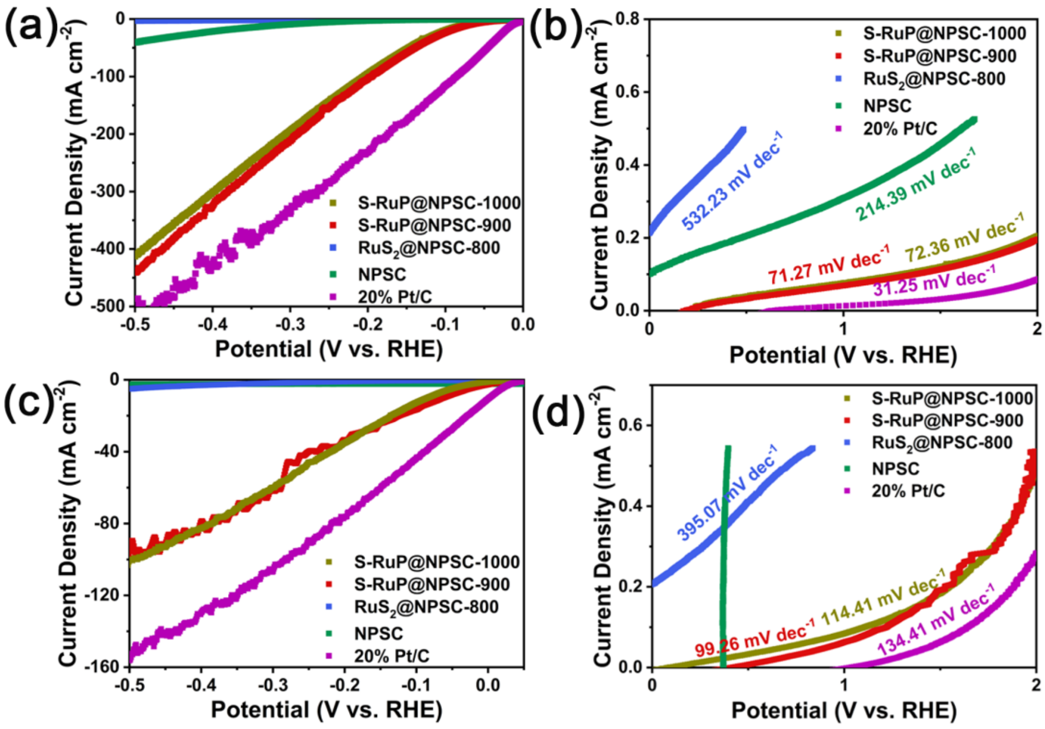

Figure S12. (a) LSV curves (b) Corresponding Tafel plots of RuS_2@NPSC-800, S-RuP@NPSC-900, S-RuP@NPSC-1000, NPSC and 20% Pt/C in 0.5 M H_2SO_4, (c) LSV curves (d) Corresponding Tafel plots of RuS_2@NPSC-800, S-RuP@NPSC-900, S-RuP@NPSC-1000, NPSC and 20% Pt/C in 1 M PBS solution, both results were without iR-corrected.

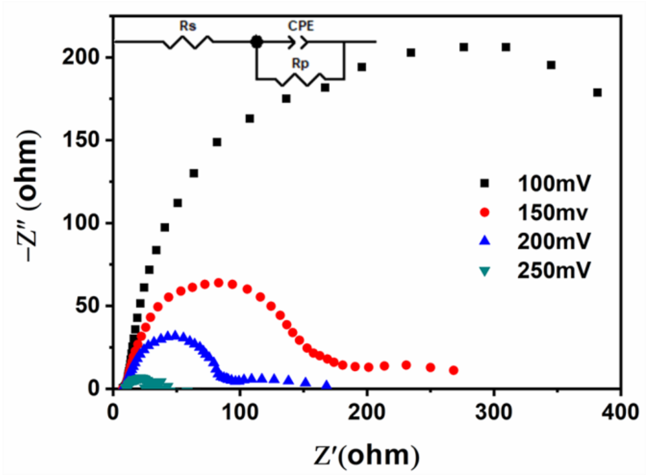

Figure S13. EIS Nyquist plots and the fitting of equivalent circuit diagram of S-RuP@NPSC-900 with different overpotentials from 100 to 250 mV.

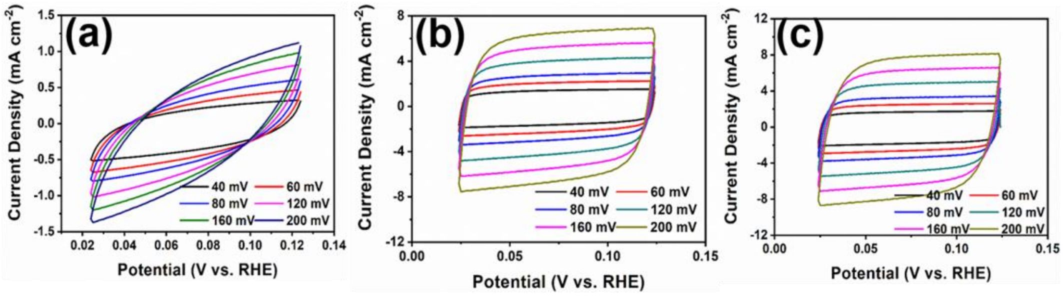

Figure S14. The cyclic voltammograms (CVs) curves of (a) RuS_2@NPSC-800, (b) S-RuP@NPSC-900, and (c) S-RuP@NPSC-1000.

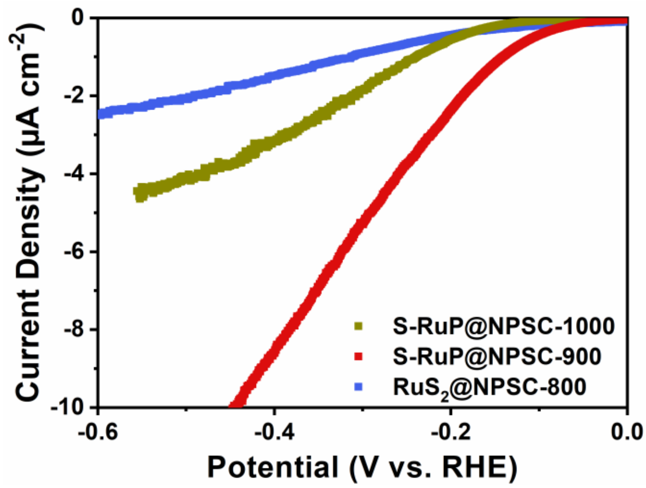

Figure S15. Polarization curves of RuS_2@NPSC-800, Ru-RuP@NPSC-900, and S-RuP@NPSC-1000 in 1 M KOH normalized by the respective electrochemical surface area.

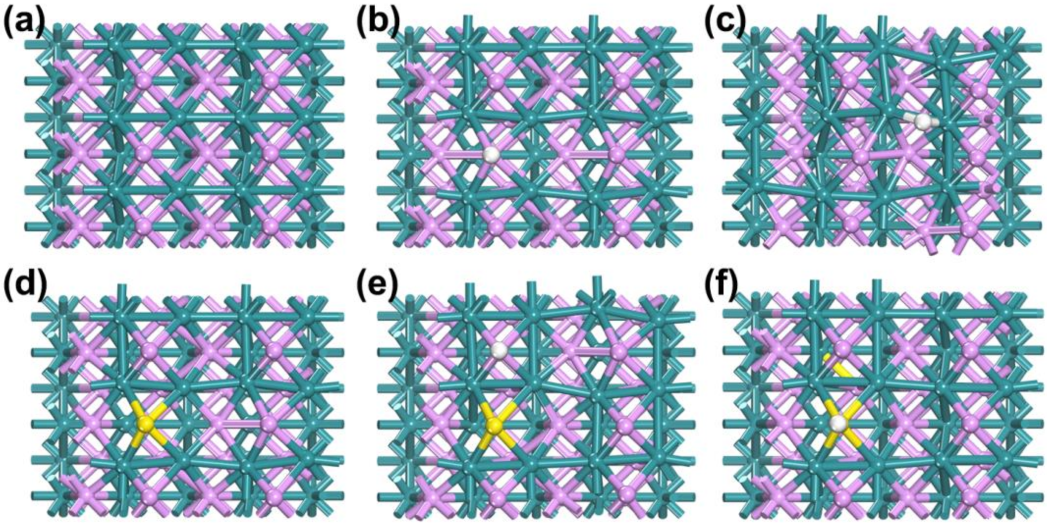

Figure S16. Optimized structure of pure RuP and S doped RuP with H* adsorption. (a) optimized structure of pure RuP, and H* adsorption on (b) Ru (c) P, (d) optimized structure of S doped RuP and H* adsorption on (e) P and (f) S in S-RuP.

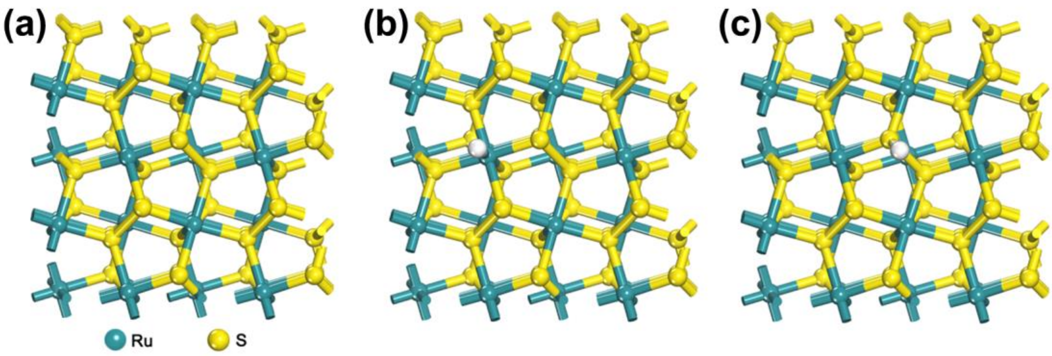

Figure S17. Optimized structure of pure RuS_2 and H* adsorption. (a) optimized structure of pure RuS_2, (b) H* adsorption on Ru and (c) H* adsorption on S.

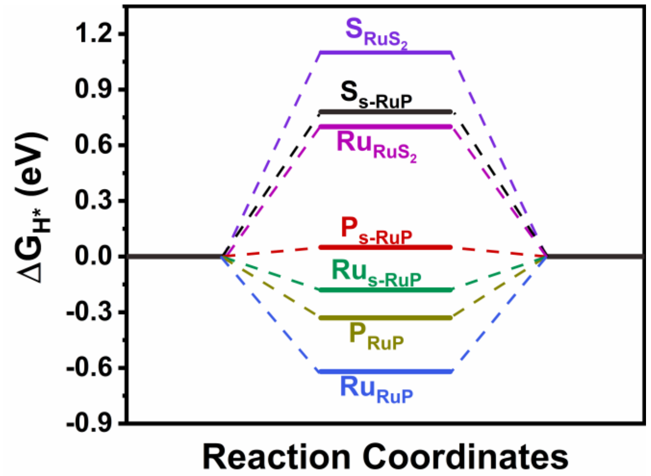

Figure S18. The Gibbs free energy of H* adsorption (ΔG_{H^*}) on various sites.

The free energy ΔG_{H^*} for chemisorbed hydrogen was computed as:

$\Delta G_{H^*} = E(total) - E(surf) - ½E(H_2) + \Delta ZPE - T\Delta S$

E(total), E(surf), ΔZPE, T, ΔS represent total energy of the adsorbed system, energy of the pure surface, change in zero-point-energy, the temperature, and change in entropy, respectively. Here, the vibrational entropy of H in the adsorbed state is approximately negligible such that $\Delta S(H) \approx [S(_{H^*}) - ½\ S(H_2)] \approx [-\ ½\ S(H_2)]$, where $[-\ ½S(H_2)]$ denotes the entropy of hydrogen in the gas phase under standard conditions. The standard $TS(H_2)$ of H_2 is given to be ~0.41 eV at 1 atm and 300 K.
